# Updating the elite rice variety Kongyu 131 by improving the *Gn1a* locus

**DOI:** 10.1186/s12284-017-0174-1

**Published:** 2017-07-20

**Authors:** Xiaomin Feng, Chen Wang, Jianzong Nan, Xiaohui Zhang, Rongsheng Wang, Guoqiang Jiang, Qingbo Yuan, Shaoyang Lin

**Affiliations:** State Key Laboratory of Plant Genomics, Institute of Genetics and Developmental Biology, University of Chinese Academy of Sciences, No.3 South Zhongguancun Road, Haidian District, Beijing, 100190 People’s Republic of China

**Keywords:** Rice, Yield, SNP marker, *Gn1a*, Qtl, Linkage drag

## Abstract

**Background:**

Kongyu 131 is an elite *japonica* rice variety of Heilongjiang Province, China. It has the characteristics of early maturity, superior quality, high yield, cold tolerance and wide adaptability. However, there is potential to improve the yield of Kongyu 131 because of the relatively few grains per panicle compared with other varieties. Hence, we rebuilt the genome of Kongyu 131 by replacing the *GRAIN NUMBER1a* (*Gn1a*) locus with a high-yielding allele from a big panicle *indica* rice variety, GKBR. High-resolution melting (HRM) analysis was used for single nucleotide polymorphism (SNP) genotyping.

**Results:**

Quantitative trait locus (QTL) analysis of the BC_3_F_2_ population showed that the introgressed segment carrying the *Gn1a* allele of GKBR significantly increased the branch number and grain number per panicle. Using 5 SNP markers designed against the sequence within and around *Gn1a*, the introgressed chromosome segment was shortened to approximately 430 Kb to minimize the linkage drag by screening recombinants in the target region. Genomic components of the new Kongyu 131 were detected using 220 SNP markers evenly distributed across 12 chromosomes, suggesting that the recovery ratio of the recurrent parent genome (RRPG) was 99.89%. Compared with Kongyu 131, the yield per plant of the new Kongyu 131 increased by 8.3% and 11.9% at Changchun and Jiamusi, respectively.

**Conclusions:**

To achieve the high yield potential of Kongyu 131, a minute chromosome fragment carrying the favorable *Gn1a* allele from the donor parent was introgressed into the genome of Kongyu 131, which resulted in a larger panicle and subsequent yield increase in the new Kongyu 131. These results indicate the feasibility of improving an undesirable trait of an elite variety by replacing only a small chromosome segment carrying a favorable allele.

## Background

Rice is one of the most important cereal crops worldwide, and more than half of the global population depends on rice as a staple food. The continuously increasing population, decreasing arable land, global climate change and expanding demands for biofuels have challenged global food security (Takeda and Matsuoka 2008). During the past 60 years, the green revolution marked by the application of semi-dwarf genes in rice (Sasaki et al. 2002; Spielmeyer et al. 2002) and the application of hybrids in southeastern Asia have greatly improved rice yield. However, research has shown that the top three rice producers, India, China and Indonesia, experienced yield stagnation in more than 37%, 78% and 81% of their respective rice growing areas from 1961 to 2008 (Ray et al. 2012). Therefore, it is challenging, but there is also great potential, to increase rice yield using molecular breeding methods in the coming decades.

The grain yield of rice is a complex quantitative trait determined by three components: effective tillers per plant, grain number per panicle and grain weight (Sakamoto and Matsuoka 2008; Xing and Zhang 2010). Grain number per panicle is further composed of two subcomponents: spikelet number, which is mainly determined by the number of primary and second branches, and the seed setting rate of the spikelets (Xing and Zhang 2010). Since the rice genome was sequenced (Goff et al. 2002; International Rice Genome Sequencing Project 2005; Yu et al. 2002), significant advancements have been made in the functional genomics of this crop. In recent years, a number of genes and quantitative trait loci (QTLs) for yield-related traits were identified and characterized (Chen et al. 2013). Major QTLs for yield traits, such as *Gn1a* (Ashikari et al. 2005), *Ghd7* (Xue et al. 2008), *GS3* (Fan et al. 2006), *GW5* (Weng et al. 2008), and *GW7* (Wang et al. 2015), have been characterized. For instance, *Gn1a* encodes cytokinin oxidase/dehydrogenase (OsCKX2), an enzyme that degrades cytokinin. When the expression of *OsCKX2* is reduced, cytokinin accumulates in inflorescence meristems and increases the number of reproductive organs, which enhances the number of grains and leads to increased grain yield (Ashikari et al. 2005). These genes, together with QTLs and their superior allelic variations, provide the basis for rice molecular breeding.

The rapid accumulation of rice genome resequencing data not only assisted in the identification of functional genes or QTLs but also provided numerous polymorphic genome sequences for molecular marker development (Huang et al. 2010, 2011, 2012; Xu et al. 2011). High-resolution markers, including restriction site-associated DNA (RAD) and single nucleotide polymorphisms (SNPs), together with the analysis platforms, improve the efficiency of genotyping and reduce the cost (Miller et al. 2007; Simko 2016; Tung et al. 2010). Based on sexual hybridization, traditional variety breeding depends on genetic recombination and phenotypic selection but has the disadvantages of low selection efficiency of complex traits, vulnerability to environmental influence and a long breeding cycle. Marker-assisted selection (MAS) involves the transformation of phenotypic to genotype selection, which can improve the efficiency and accuracy of the selection of target traits, and has been widely used for crop genetic improvement (Chen et al. 2013; Jena and Mackill 2008; Xu and Crouch 2008).

Breeders have improved the resistance to rice blast, bacterial blight and brown planthopper with the aid of MAS (Jiang et al. 2012; Liu et al. 2016; Pradhan et al. 2015; Wang et al. 2016). Although MAS has been applied in the breeding of many crops, it has limitations. First, MAS selects the target traits through the molecular markers linked to the genes for the target traits, and the recombination between the molecular markers and target genes can reduce the selection accuracy. To date, almost all research on resistance breeding through MAS used markers linked to resistance genes, but reports on the selection of target genes through genic or functional markers (Andersen and Lubberstedt 2003) are rare. Second, introducing target genes through MAS can result in linkage drags. Third, introducing beneficial allelic genes cannot efficiently improve the target traits due to different genomic backgrounds. Solving these problems can make molecular breeding more accurate and efficient. In addition, there are many reports on improvements in disease and insect resistance, but reports on complex traits, such as yield improvement, are rare.

Kongyu 131 ranked top among the elite rice varieties of the third accumulative temperature belt in Heilongjiang Province because of its superior traits such as early maturity, superior quality, cold tolerance and wide adaptability. In 2005, the planting area of Kongyu 131 reached 770,000 ha, accounting for more than half of the total rice planting area. We improved the resistance of Kongyu 131 to rice blast by improving its blast resistance gene locus (Zhang et al. 2017). We also found that Kongyu 131 has the disadvantage of relatively few grains per panicle compared with other varieties; therefore, the objective of this study was to improve the grain number per panicle and subsequent yield of Kongyu 131 by substituting the *Gn1a* locus in the genome of Kongyu 131 with a favorable allele from GKBR.

## Results

### Comparison of *Gn1a* sequences of the rice parents

**Fig. 1:**
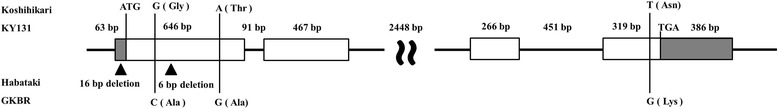
*Gn1a* structure and allelic variation of Kongyu 131, GKBR, Koshihikari and Habataki. Three SNPs in the coding sequence that resulted in amino acid changes (in parentheses) were identified and are shown as black vertical lines. A 16-bp deletion in the 5′-untranslated region and a 6-bp deletion in the first exon were identified and are shown as triangles. Gray and white rectangles and black horizontal lines represent untranslated regions, exons and introns, respectively

The *Gn1a* sequences of Kongyu 131, GKBR, Koshihikari and Habataki were compared. Kongyu 131 had a coding DNA sequence (CDS), 5′-untranslated region (5′-UTR) and 3′-untranslated region (3′-UTR) of *Gn1a* identical to Koshihikari, and the CDS, 5′-UTR and 3′-UTR of *Gn1a* from GKBR was the same as that from Habataki. Several nucleotide changes, including a 16-bp deletion in the 5′-UTR, a 6-bp deletion in the first exon, and three nucleotide changes resulting in amino acid variation in the first and fourth exons of the GKBR allele, were revealed (Fig. 1).

### Genotyping

**Fig. 2:**
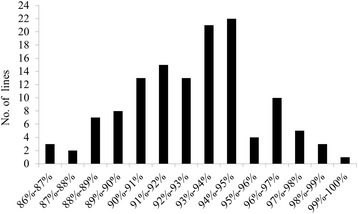
Distribution of the recovery ratio of the recurrent parent genome in the BC_3_F_1_ population

**Fig. 3:**
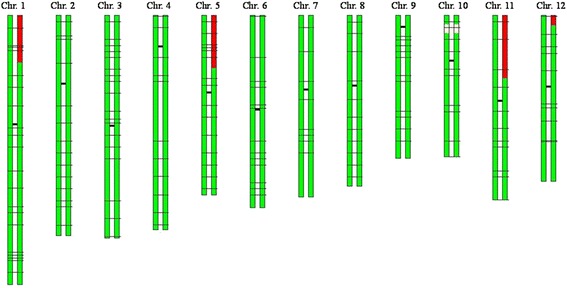
Graphical genotype (GGT) of BC_3_F_1_-22F01. Green bars represent the chromosome fragments derived from Kongyu 131, red bars represent the chromosome fragments derived from GKBR, and white bars represent unknown fragments; the black horizontal lines indicate the SNP markers (the shorter, darker lines represent the centromeres)

One-hundred-and-sixty SNP markers evenly distributed over 12 chromosomes were used to genotype the BC_3_F_1_ population consisting of 127 lines. The RRPG ranged from 86.3% to 99.5% with an average of 92.83% (Fig. 2). This value is close to the theoretical RRPG of 93.75% in the BC_3_F_1_ generation, and the difference could be attributed to unidentified genotypes of some markers in certain lines. The line named BC_3_F_1_-22F01 carried 4 fragments of GKBR on chromosome 1, 5, 11 and 12, according to the 160 SNP markers, and the RRPG was 96.27% (Fig. 3).

### Performance of the traits in Kongyu 131 and the BC_3_F_2_-LQ96 population

Panicle length (PL, cm), number of primary branches per panicle (NPB), grain number per panicle (GNP), density of the primary branches (DPB, /cm) and grain density per panicle (GDP, /cm) in Kongyu 131 and the BC_3_F_2_-LQ96 population are shown in Table 1. The means of the above five traits in the BC_3_F_2_-LQ96 population were higher than those of Kongyu 131, which indicated that the chromosome fragments derived from GKBR could increase the phenotypic value of yield-related traits.

**Table 1:** Performance of the traits in Kongyu 131 and the random subpopulation BC_3_F_2_-LQ96 derived from BC_3_F_1_-22F01 using Kongyu 131 as the recurrent parent and GKBR as the donor parent

Traits	KY131	BC_3_F_2_-LQ96 population	
	Mean ± SD	Mean ± SD	Range
PL (cm)	16.8 ± 0.3	17.4 ± 1.5	14.7 ~ 20.1
NPB	12.8 ± 0.4	15.2 ± 1.8	12.0 ~ 19.0
DPB (/cm)	0.76 ± 0.02	0.88 ± 0.10	0.73 ~ 1.22
GNP	120.4 ± 5.9	172.4 ± 29.0	113.0 ~ 235.0
GDP (/cm)	7.16 ± 0.37	9.91 ± 0.20	7.09 ~ 13.81

### Correlation analysis of the yield-related traits

Table 2 shows the correlation coefficients among the 5 yield-related traits. Positive and significant correlations were detected among these traits except for PL and DPB (*r* = −0.059). The strongest correlation was detected between GNP and GDP (*r* = 0.914), whereas the correlation between DPB and GNP was relatively low (*r* = 0.355).

**Table 2:** Correlation coefficients of the five yield-related traits investigated in the random subpopulation BC_3_F_2_-LQ96 derived from BC_3_F_1_-22F01 using Kongyu 131 as the recurrent parent and GKBR as the donor parent

	PL	NPB	DPB	GNP
NPB	0.598^**^			
DPB	−0.059	0.763^**^		
GNP	0.768^**^	0.777^**^	0.355^*^	
GDP	0.459^**^	0.721^**^	0.533^**^	0.914^**^

^*^Significant at p<0.05; significant at ^**^p<0.01

### Marker-trait association analysis

**Fig. 4:**
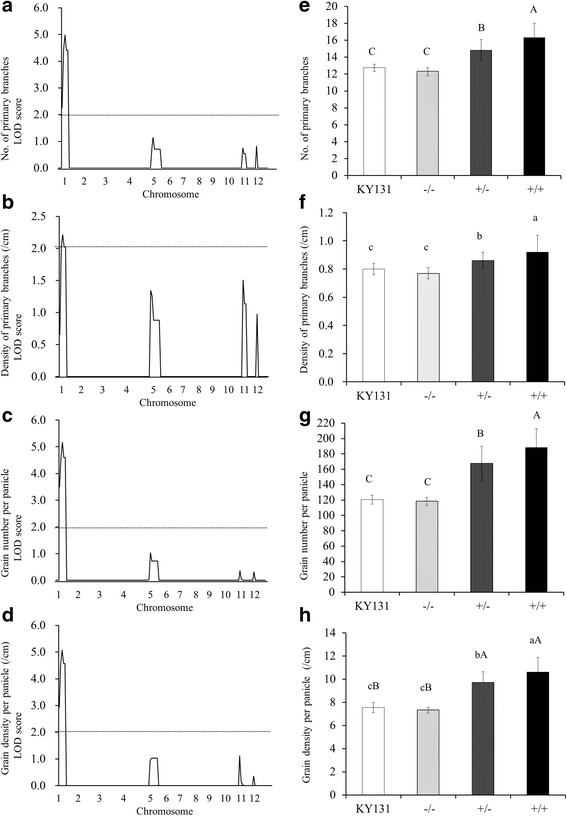
Marker-trait analysis of the random subpopulation BC_3_F_2_-LQ96 derived from BC_3_F_1_-22F01 using Kongyu 131 as the recurrent parent and GKBR as the donor parent. **a**-**d** LOD score of 4 yield-related traits; the horizontal axis represents the 160 SNP markers on 12 chromosomes. **e**-**h** Comparison of 4 yield-related traits of Kongyu 131 and three genotypic classes of the *Gn1a* locus. −/− represents homozygote for Kongyu 131, +/− represents heterozygote, and +/+ represents homozygote for GKBR. **a** and **e** Number of primary branches per panicle (NPB). **b** and **f** Density of primary branches (DPB, /cm). **c** and **g** Grain number per panicle (GNP). **d** and **h** Grain density per panicle (GDP, /cm). **e**-**h** A, B, C ranked by Student’s t-tests at *P* ≤ 0.01; a, b, c, ranked by Student’s t-tests at *P* ≤ 0.05

Genetic analysis of the BC_3_F_2_-LQ96 population showed that the fragment located on chromosome 1 had significant and simultaneous effects on NPB, DPB, GNP and GDP (Fig. 4a-d). The interval SNP1-SNP5 containing the *Gn1a* locus contributed to 39.9%, 20.3%, 41.1% and 40.4% of the phenotypic variation of these traits, respectively (Table 3). The allele from GKBR contributed to the increases in NPB, DPB, GNP and GDP with additive effects of 2.0, 0.08, 34.95 and 1.65, respectively. This was consistent with these traits beingstrongly correlated. In addition, partial dominance was observed for these traits. The mean NPB, DPB, GNP and GDP values of Kongyu 131 and the three genotypic classes at the *Gn1a* locus (homozygote for Kongyu 131, heterozygote, homozygote for GKBR) were compared (Fig. 4e-h). The average phenotypic values of the homozygote for GKBR and the heterozygote were significantly higher than those of Kongyu 131, but no difference was observed between Kongyu 131 and the homozygote for Kongyu 131. In addition, the phenotypic value of the homozygote for GKBR was significantly higher than that of the heterozygote. These results show that the introgressed GKBR chromosome fragment carrying the favorable *Gn1a* allele significantly increased NPB, DPB, GNP, and GDP, and the increase in GNP was mainly due to the NPB as opposed to the PL.

**Table 3:** Effects of the QTL (in the interval SNP1-SNP5) on yield-related traits detected in the random subpopulation BC_3_F_2_-LQ96 derived from BC_3_F_1_-22F01 using Kongyu 131 as the recurrent parent and GKBR as the donor parent

Traits	Chr	Nearest	Additive	Dominant	LOD	Var.%
		marker	effect	effect		
NPB	1	SNP3	2.00	0.50	5.0	39.9%
DPB	1	SNP3	0.08	0.02	2.2	20.3%
GNP	1	SNP3	34.95	14.05	5.2	41.1%
GDP	1	SNP3	1.65	0.74	5.1	40.4%

Var.% represents the percentage of total phenotypic variance explained by the QTL

### Evaluation of the selected lines

**Fig. 5:**
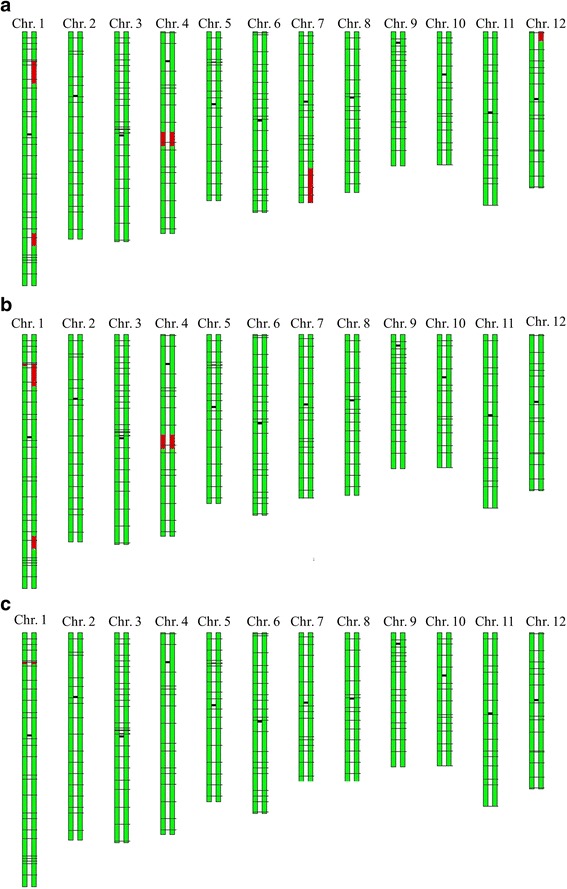
GGT of the selected lines. **a** BC_3_F_2_-2B09. **b** BC_3_F_3_-652E09. **c** BC_4_F_2_-350C05. The green bars represent the chromosome fragments derived from Kongyu 131, the red bars represent the chromosome fragments derived from GKBR, and the horizontal black lines indicate the SNP markers(the shorter, darker lines represent the centromeres)

Two-hundred-and-twenty SNP markers evenly distributed across the genome were used to genotype the line BC_3_F_2_-2B09. The average distance between adjacent markers was 1.7 Mb. Owing to the crossover between SNP1 and SNP2 in the region upstream of *Gn1a,* the target chromosome fragment was shortened to approximately 4 Mb. There were 4 non-target fragments on chromosomes 1, 4, 7 and 12, among which the fragments at the end of the long arm of chromosomes 1, 4 and 7 were detected by adding 60 SNP markers located in the large interval without markers. The RRPG of BC_3_F_2_-2B09 was 97.49% based on the 220 SNP markers (Fig. 5a). To further shorten the target chromosome fragment, BC_3_F_3_-652E09 produced from a crossover between SNP4 and SNP5 was selected, and the target chromosome fragment was shortened to approximately 430 Kb. In addition, two non-target fragments were observed on chromosomes 1 and 4. The RRPG of BC_3_F_3_-652E09 was 98.45% (Fig. 5b). Finally, the improved line named BC_4_F_2_-350C05 with the same genome as Kongyu 131 except near the *Gn1a* locus was developed. The RRPG of BC_4_F_2_-350C05 was 99.89% (Fig. 5c).

### Evaluation of the agronomic traits of Kongyu 131 and the improved line

**Fig. 6:**
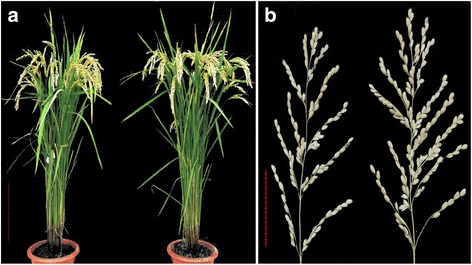
Plant type and the main panicle of Kongyu 131 and its improved line BC_4_F_2_-350C05 at Changchun. **a** Plant type. Kongyu 131 (left), BC_4_F_2_-350C05 (right); scale bar represents 20 cm. **b** Main panicle. Kongyu 131 (left), BC_4_F_2_-350C05 (right); scale bar represents 5 cm

The plant type and main panicle of Kongyu 131 and its improved line BC_4_F_2_-350C05 are shown in Fig. 6, and the performance of the agronomic traits at the two locations is presented in Table 4. At Changchun, the DTH of BC_4_F_2_-350C05 was 4 days longer than Kongyu 131, while no significant difference was detected at Jiamusi, indicating that the DTH is vulnerable to the environment. Compared with Kongyu 131, the PH of BC_4_F_2_-350C05 increased by 4.0 cm and 5.4 cm at Changchun and Jiamusi, respectively. The YP of BC_4_F_2_-350C05 increased by 4.7 g and 6.9 g at Changchun and Jiamusi, i.e., increases of 8.3% and 11.9%, respectively. The lower YP response at Changchun can be explained by the fewer ETP and GNP and lower SSP. The NPB, DPB, GNP and GDP of BC_4_F_2_-350C05 were significantly higher than those of Kongyu 131 at both Changchun and Jiamusi, which contributed to the increase in YP. The reduced GT caused a reduction (1.3 g and 2.0 g) in the TGW of BC_4_F_2_-350C05 at Changchun and Jiamusi, respectively, compared with Kongyu 131. However, the ETP, PL, GL, GW and LWR of BC_4_F_2_-350C05 at the two locations were similar to those of Kongyu 131.

**Table 4:** Agronomic performance of Kongyu 131 and its improved line BC_4_F_2_-350C05 at two locations

Traits	Changchun		Jiamusi	
	KY131	BC_4_F_2_-350C05	KY131	BC_4_F_2_-350C05
DTH	93.2 ± 0.8	97.2 ± 1.5^**^	103.6 ± 0.8	103.1 ± 0.7
PH (cm)	70.7 ± 2.2	74.7 ± 1.1^**^	70.6 ± 1.8	76.0 ± 3.1^**^
ETP	27.3 ± 3.3	27.1 ± 3.1	31.5 ± 3.6	31.4 ± 2.4
PL (cm)	16.6 ± 0.5	17.4 ± 0.5	16.5 ± 0.9	17.2 ± 0.4
NPB	11.8 ± 0.6	14.7 ± 0.6^**^	12.2 ± 1.0	14.9 ± 0.5^**^
DPB (/cm)	0.71 ± 0.04	0.85 ± 0.06^**^	0.75 ± 0.09	0.86 ± 0.02^**^
GNP	119.3 ± 8.0	168.1 ± 2.6^**^	123.4 ± 8.3	186.2 ± 17.8^**^
GDP (/cm)	7.18 ± 0.45	9.79 ± 0.11^**^	7.50 ± 0.38	10.69 ± 0.79^**^
SSP (%)	97.0 ± 1.7	93.8 ± 1.9^**^	98.0 ± 0.3	97.4 ± 0.6
YP (g)	56.4 ± 1.1	61.1 ± 1.6^**^	57.8 ± 1.1	64.7 ± 1.8^**^
TGW (g)	27.6 ± 0.6	26.3 ± 0.6^**^	27.8 ± 0.0	25.8 ± 0.3^**^
GL (mm)	7.4 ± 0.2	7.6 ± 0.1	7.4 ± 0.1	7.3 ± 0.1
GW (mm)	3.6 ± 0.1	3.6 ± 0.0	3.6 ± 0.1	3.6 ± 0.1
LWR	2.06 ± 0.03	2.05 ± 0.01	2.05 ± 0.00	2.07 ± 0.02
GT (mm)	2.33 ± 0.00	2.28 ± 0.04^**^	2.33 ± 0.02	2.30 ± 0.01^*^
TYP (Kg)	5.41 ± 0.26	5.87 ± 0.32^**^	5.55 ± 0.31	6.21 ± 0.43^**^
AYP (Kg)	4.38 ± 0.21	4.72 ± 0.26^**^	4.55 ± 0.25	5.01 ± 0.35^**^

Data presented as the means with standard deviations were obtained from plants in a randomized complete block design with three replications under natural conditions in Changchun and Jiamusi in 2016. The planting density was 30 cm × 20 cm, with one plant per hill. AYP data were obtained from plants in 10 plots, and the area per plot was 5.76 m ^2^(2.4 m × 2.4 m)

^*^represents significance at *p* ≤ 0.05 based on Student’s t-tests; ^**^ represents significance at *p* ≤ 0.01 based on Student’s t-tests

## Discussion

As a preeminent rice variety, even though Kongyu 131 has potential for increased production, it is difficult to improve yield while maintaining other desirable traits using MAS. In this study, the genome of Kongyu 131 was rebuilt through crossing, backcrossing and self-crossing. During this process, non-target chromosome fragments were excluded by background selection, and linkage drag was minimized by recombinant selection from large populations. Subsequently, a new Kongyu 131 genome carrying the favorable allele of *Gn1a* was built. We termed this method “Variety Update”, which can be used to improve a less desirable trait of an elite variety by replacing only a minute chromosome fragment (Lin et al. 2009).

### Limitations of MAS and solutions in this study

The recombination between the target gene and linked markers would affect the selection accuracy through MAS. In this study, the genic marker SNP3 was developed according to the polymorphic *Gn1a* sequence between Kongyu 131 and GKBR. Genic markers derived from target gene sequences co-segregate with the target gene, which can effectively prevent recombination between markers and genes for a target trait (Andersen and Lubberstedt 2003). Moreover, linkage drag often occurs during MAS (Lewis et al. 2007). To minimize possible linkage drag, we developed SNP1, SNP2, SNP4 and SNP5 around the *Gn1a* locus, aiming to shorten the target introgressed chromosome fragment by screening the recombinant lines between SNP1 and SNP2 as well as between SNP4 and SNP5.

Introducing beneficial allelic genes through MAS may not improve the target traits efficiently due to different genomic backgrounds. In this study, Kongyu 131 was used as the recurrent parent, and GKBR as the donor parent, to develop an advanced backcrossed population. Advanced backcross QTL analysis (AB-QTL) (Naz et al. 2008; Sayed et al. 2012) was performed using the BC_3_F_2_ population to confirm that the introgressed fragment carrying *Gn1a* could significantly increase the branch number and grain number per panicle. Consequently, the new Kongyu 131 carrying the favorable *Gn1a* was developed.

### Size of the introgressed chromosome fragment and marker number to detect the donor’s fragments in the background

A large introgressed target chromosome fragment and the donor’s chromosomal fragments remaining in the background can lead to undesirable traits in an improved line with a new genome. To solve this problem, on the one hand, we attempted to reduce the size of the introgressed chromosome fragment with the target gene, and on the other hand, to exclude all of the donor’s chromosomal fragments from the background. In this study, the target introgressed chromosomal fragment was shortened to less than 856 Kb, and the actual length needed to be identified by adding markers between SNP1 and SNP2 as well as between SNP4 and SNP5. It remains to be determined whether this size is appropriate, but it does not matter if no adverse traits are identified in the production test. Whether there are unfavorable genes within the target introgressed chromosome fragment can be determined through a production test and also speculated theoretically. If wild rice is used as the donor parent, the introgressed chromosome fragment is more likely to carry unfavorable genes without artificial selection. Then, the target introgressed fragment should be smaller than that when cultivated rice is used as the donor parents to prevent interaction between the foreign genes and the background. Furthermore, if cultivated rice with a close genetic relationship is used as the donor parents, then a relatively large target introgressed fragment is also acceptable. In this study, 220 SNP markers evenly distributed across the genome were used to detect the donor’s chromosome fragments in the background. However, it is unclear whether this number of markers is sufficient. Both the genotyping cost and a practical production test need to be taken into account. The new variety also needs to be observed for a certain period of time, and the production test results will reflect whether this number of markers is sufficient.

### Agronomic traits in the new Kongyu 131 were improved

The YP of the improved line increased significantly at both Changchun and Jiamusi compared to Kongyu 131, and the improvement in YP was mainly because of the increase in NPB and GNP. This result indicates that incorporating the GKBR allele of *Gn1a* into the genome of Kongyu 131 increased the YP, and the improved line can be used as a new variety for rice production.

The DTH of the improved line at Jiamusi was 10 days longer, and 4 more ETP were produced compared with Changchun, which may be caused by the different photoperiods and temperatures of the two locations. The increase in the PH of the improved line, BC_4_F_2_-350C05, may be caused by the pleiotropy of *Gn1a* or undetected donor chromosome fragments. The reduced GT of the improved line caused the TGW to decrease by 1.3 g and 2.0 g at the two locations compared with Kongyu 131. This result may be explained by the fact that the GNP of the improved line increased, but the DTH did not, resulting in insufficient accumulation of photosynthetic products. It is generally believed that rice plants with a delayed heading date produce relatively more biomass than early heading plants, such as *Ghd7* (Xue et al. 2008)*, DTH8* (Wei et al. 2010)*, Hd1* (Yano et al. 2000) and *DTH7* (Gao et al. 2014). In the future, we will verify whether the yield may increase further by delaying the heading date through pyramiding certain genes for a late heading date.

### The foundation for elite variety updating

To improve the *Gn1a* locus of Kongyu 131, we rebuilt the genome of Kongyu 131 through crossing and backcrossing. The new Kongyu 131 possessed the same genome as Kongyu 131 except close to the *Gn1a* locus, based on 220 SNP markers across the whole genome. The Variety Update method will probably become an increasingly more popular approach for the following reasons: farmers in developed and developing countries prefer to grow their ‘tried and tested’ varieties. In addition, farmers have already determined the optimum sowing rates and date, fertilizer application rates and timing of irrigation events for these varieties.

QTL analysis was carried out to confirm the target traits, and the results showed that selecting target traits entirely by genotype was reliable using this method. In this study, a chromosome fragment smaller than 856 Kb from donor parents was introgressed; therefore, in addition to *Gn1a*, other genes in the vicinity were also included in the genome of Kongyu 131. Whether these genes changed the genome composition and structure of Kongyu 131 or whether there were other undetected donor chromosome fragments in the background leading to undesirable traits is still not fully understood. However, the new variety bred using this method possesses genome information; if adverse traits appear in production, we can easily trace the causative genes or donor chromosome fragments in the background. In addition, if the adverse traits are caused by the loci of Kongyu 131, this problem can be solved using the same method. Therefore, the results from this study can be compiled and used to lay the foundation for future variety update studies.

### Prospects

The elite rice variety Kongyu 131 was used as the recurrent parent, and only the *Gn1a* locus was improved to increase the grain number per panicle and subsequent yield in this study. Traits such as heading date, quality, disease and insect resistance, stress tolerance and nutrient efficiency can be improved using the same method in the future. Consequently, a series of new Kongyu 131 varieties meeting local needs could be developed by pyramiding two or more favorable genes. In addition, HRM is a method that allows polymorphism detection in double-stranded DNA by comparing the profiles of melting curves (Simko 2016). Only 6 ~ 10 min are needed to perform SNP genotyping through HRM after PCR; thus, this approach will become increasingly popular in molecular breeding.

## Conclusions

To improve the yield of the elite rice variety Kongyu 131 and reserve its other desirable agronomic traits, we attempted a new approach, i.e., the rebuilding of the Kongyu 131 genome. During the process of genome reconstruction, the favorable allele *Gn1a* from the donor parent was introgressed into the genome of Kongyu 131. The yield per plant of the new Kongyu131 increased by 8.3% and 11.9% in two locations, and this new variety can be deployed in Heilongjiang Province instead of Kongyu131.

## Methods

### Rice materials

The recurrent parent Kongyu 131, an early maturity *japonica* rice variety grown in the high latitude zone, has strong tillering ability, is lodging resistant and cold tolerant, and requires an active accumulated temperature of 2330 °C. The average yield in Heilongjiang Province is 7684.5 kg/ha. The donor parent GKBR is an *indica* rice variety selected from the progenies derived from crossing 93–11 with IR64 and from self-crossing several times. GKBR has large panicles and dense spikelets and is blast resistant. The growth period of GKBR in Guangzhou, China, is 113 days in late season, but normal heading does not occur in Heilongjiang Province due to unsuitable photoperiod and temperature conditions.

### Population development, field experiments and traits measurement

**Fig. 7:**
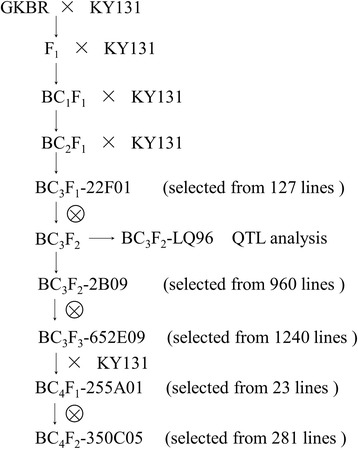
Procedure for the genome reconstruction of Kongyu 131 (KY131)

GKBR was crossed and backcrossed with Kongyu 131 to produce a BC_3_F_1_ population that included 127 lines. A line named BC_3_F_1_-22F01 with a chromosome segment carrying the *Gn1a* locus and the highest recovery ratio of the recurrent parent genome (RRPG) was selected to produce a BC_3_F_2_ population. From the BC_3_F_2_ population, 96 plants were randomly selected to form a BC_3_F_2_-LQ96 subpopulation for QTL analysis (Fig. 7). The BC_3_F_2_-LQ96 population was planted at the experimental farm of Jiamusi (130°57′E, 46°23′N), Heilongjiang Province, in the rice-growing season of 2015. Sowing and raising seedlings were conducted in a 96-well polymerase chain reaction (PCR) plate with a hole (Φ = 2.5 mm) at the bottom; one seed was placed in each well with the embryo pointing upward, and seedlings at the 3 ~ 4 leaf stage were transplanted to a paddy field. Each plot included 8 rows with 12 plants in each row; the plant spacing was 20 cm within rows and 30 cm between adjacent rows. The field management was the same as the normal local paddy field. At harvesting, the panicle length (PL, cm), number of primary branches per panicle (NPB) and grain number per panicle (GNP) were measured. The density of primary branches (DPB, /cm) and grain density per panicle (GDP, /cm) were calculated as DPB = NPB / PL and GDP = GNP / PL.

### Resequencing, comparison of *Gn1a* and marker design

The Kongyu 131 and GKBR rice varieties were sequenced using a HiSeq 2000 sequencing system. We downloaded the *Gn1a* DNA sequence of Koshihikari and Habataki (Ashikari et al. 2005) from GenBank (www.ncbi.nlm.nih.gov/genbank) and compared the *Gn1a* DNA sequences of Kongyu 131, GKBR, Koshihikari and Habataki using DNAMAN. SNP markers were developed according to the SNPs between Kongyu 131 and GKBR. The SNPs and their flanking 22 ~ 24- nt special sequences were used to design locus-specific and allele-specific primers; the amplicons were approximately 50 ~ 100 bp. Five SNP markers named SNP1-SNP5 were designed(Table 5). SNP3 was located in *Gn1a*, SNP1 and SNP2 were located upstream of *Gn1a*, while SNP4 and SNP5 were located downstream of *Gn1a.* SNP2 was close to the 5′-UTR of *Gn1a* and SNP4 was close to the 3′-UTR of *Gn1a.* The distance of SNP2 and SNP4 was 5761 bp, while the distance between SNP1 and SNP5 was 856 Kb.

**Table 5:** Sequences of the SNP markers developed for the selection of *Gn1a*

Markers	Chr	Position	Forward primer	Reverse primer
SNP1	1	5,006,541	ATGCGTGTGGCCCTTGAAAATG	AGATCTTCAAGGACGATTAAG
SNP2	1	5,269,396	ATTCAAGCATGCCGTACGTTTG	AGCCTTCATATGCATGTCGATC
SNP3	1	5,272,491	TCCAAAACAGTGAAAAGCATGC	TCTAGCTACTACCTACACTAGC
SNP4	1	5,275,157	ACTTGGGCCTAATGGCTAGCAG	TAGGGTGGCTATACTAACCAGT
SNP5	1	5,862,787	AGCATGCAAATAACGAGATGTC	CTATTTTAAATTCTTGAGAGGT

Position refers to the physical location of IRGSP-1.0

### DNA extraction, PCR and HRM analysis

Genomic DNA was extracted from fresh rice leaves following the high-throughput protocol of plant genomic DNA preparation (Wang et al. 2013). PCR reactions were performed using a 96-well plate with a 10-μL reaction mixture that contained approximately 50 ng genomic DNA, 1 μL 10× Easy Taq buffer (Transgen Biotech Inc., Beijing, China), 0.2 μL 2.5 mM dNTPs (Transgen Biotech Inc., Beijing, China), 0.5 U Easy Taq DNA Polymerase (Transgen Biotech Inc., Beijing, China), and 0.125 μL 20× EvaGreen (Biotium Inc., USA); the reactions were covered with 10 μL mineral oil (Amresco Inc., USA). The PCR amplification program consisted of one cycle of denaturation at 95 °C for 5 min, followed by 40 cycles at 95 °C for 10 s and 55°Cfor 30 s. Subsequently, the PCR plates were subjected to HRM analysis using a LightCycler®96 (Roche Inc., Swit), SNP genotyping analysis using HRM was carried out as described by Hofinger et al. (2009).

### Data analysis

A total of 160 SNP markers distributed evenly over 12 chromosomes were used to genotype the BC_3_F_1_ population, and heterozygous markers were used to detect the BC_3_F_2_-LQ96 population. QTL analysis was performed using Mapmaker/QTL 1.1b (Lincoln et al. 1992) at a threshold LOD of 2.0. Correlation analysis among traits was conducted using SAS 9.3.

### Selecting the line with the improved genome

To shorten the introgressed target chromosome fragment and to minimize linkage drag, 90 recombinant lines with crossovers between SNP1 and SNP5 were selected from 960 BC_3_F_2_ plants derived from BC_3_F_1_-22F01. Then, the markers heterozygous for BC_3_F_1_-22F01 and 60 newly added markers located in the large interval without markers were used to genotype the 90 recombinant lines, from which a line named BC_3_F_2_-2B09, heterozygous at SNP2-SNP4 but homozygous Kongyu 131-type at SNP1 and with the highest RRPG, was selected (Fig. 7). Second, 20 lines with the chromosome fragment carrying GKBR’s *Gn1a* allele and crossing over between SNP4 and SNP5 were selected from 1240 BC_3_F_3_ plants derived from BC_3_F_2_-2B09. Then, the markers heterozygous for BC_3_F_2_-2B09 were used to genotype the 20 recombinant lines, from which a line named BC_3_F_3_-652E09 with the highest RRPG was selected (Fig. 7). Third, BC_3_F_3_-652E09 was crossed with Kongyu 131 to develop the BC_4_F_1_ population consisting of 23 lines, among which a line named BC_4_F_1_-255A01 was selected. From 281 BC_4_F_2_ plants derived from BC_4_F_1_-255A01, the improved line BC_4_F_2_-350C05 with the homozygous GKBR chromosome fragment at the *Gn1a* locus and excluding other non-target fragments was selected (Fig. 7). The genome of BC_4_F_2_-350C05 was the same as Kongyu 131 except in the vicinity of the *Gn1a* locus according to the 220 SNP markers.

### Evaluation of agronomic traits

The improved line and Kongyu 131 were planted at Changchun (125°18′E, 44°43′N), Jilin Province, and at Jiamusi with three replications. Each replication consisted of 8 rows with 12 plants per row. At the time of harvest, 10 plants in the middle of each plot were selected randomly to investigate the phenotypic data; every selected plant had to meet the condition that its surrounding 8 plants exhibited normal growth vigor. Days to heading (DTH) were calculated from the time of seed soaking to when 50% of the plants flowered in the plots. Plant height (PH), effective tillers per plant (ETP), panicle length (PL), number of primary branches per panicle (NPB), grain number per panicle (GNP), grain length (GL), grain width (GW), grain thickness (GT), thousand grain weight (TGW), yield per plant (YP) and the moisture content of the grain were investigated. The yield per plant at a moisture content of 15%, the density of primary branches (DPB), grain density per panicle (GDP), seed setting percentage (SSP) and grain length to width ratio (LWR) were calculated.

## Abbreviations

AYP: Actual yield per plot
CDS: Coding DNA sequence
DPB: Density of primary branches
DTH: Days to heading
ETP: Effective tillers per plant
GDP: Grain density per panicle
GGT: Graphical genotype
GL: Grain length
GNP: Grain number per panicle
GT: Grain thickness
GW: Grain width
HRM: High-resolution melting
LWR: Length to width ratio
MAS: Marker-assisted selection
NPB: Number of primary branches per panicle
PH: Plant height
PL: Panicle length
QTL: Quantitative trait loci
RAD: Restriction site-associated DNA
RFLP: Restriction fragment length polymorphism
RRPG: Recovery ratio of recurrent parent genome
SNP: Single nucleotide polymorphism
SSP: Seed setting percentage
SSR: Simple sequence repeat
TGW: Thousand grain weight
TYP: Theoretical yield per plot
UTR: Un-translated regions
YP: Yield per plant
